# Efficacy and Blood Levels of Lacosamide in Patients with Focal Epilepsy

**DOI:** 10.3390/jcm13226958

**Published:** 2024-11-19

**Authors:** Toshiyuki Iwasaki, Toshihiro Kobayashi, Yusaku Miyamoto, Taichi Imaizumi, Shotaro Kaku, Noriko Udagawa, Hitoshi Yamamoto, Naoki Shimizu

**Affiliations:** 1 Department of Pediatrics, Kawasaki Municipal Tama Hospital, Kawasaki 214-8522, Kanagawa, Japan; t2imaizumi@marianna-u.ac.jp (T.I.); s3kaku@marianna-u.ac.jp (S.K.); 2 Department of Pediatrics, St. Marianna University School of Medicine, Kawasaki 216-8511, Kanagawa, Japan; yusaku@marianna-u.ac.jp (Y.M.); n2kami@marianna-u.ac.jp (N.U.); h3yama@marianna-u.ac.jp (H.Y.); naoki.shimizu@marianna-u.ac.jp (N.S.); 3 Department of Pharmacy, Kawasaki Municipal Tama Hospital, Kawasaki 214-8522, Kanagawa, Japan; toshihiro.kobayashi@marianna-u.ac.jp

**Keywords:** blood level, lacosamide, optimal range, reduction in epileptic seizure rate, therapeutic drug monitoring

## Abstract

**Objectives**: The aim of this paper is to analyze clinical targets for lacosamide (LCM) blood levels in patients with focal epilepsy. Referring to the LCM optimal range will encourage us to think about the importance and usefulness of measuring its blood levels. **Methods**: A total of 101 (45 female, 56 male) patients were treated with LCM. Blood sampling was performed 1 month after the start of oral medication (the levels reached a steady state) if the LCM treatment had been continued, and then 6 and 12 months after. The efficacy of LCM was evaluated by the reduction in the epileptic seizure rate (RR) at the time of blood sampling. The patients were classified as effective cases (seizure reduction rate ≥ 50%) and ineffective cases (<50%). The actual level, the calculated peak/trough levels, and the levels for each type of seizure were investigated. A statistical analysis was performed using Spearman’s rank correlation coefficient and the Wilcoxon signed-rank test. **Results**: A positive correlation was seen between blood levels and dosage (r = 0.446). However, the blood levels and RR showed no correlation. The blood levels were higher in effective cases than in ineffective cases at all time points (measurement *p* < 0.001, peak *p* = 0.013, trough *p* = 0.001). Because the range was set so that the effective and ineffective groups did not overlap, the optimal range of LCM was found to be 8.0–10.5 µg/mL. **Conclusions**: Measuring and calculating blood levels of LCM and adjusting the dosage to reach the optimal range are recommended. Moreover, the optimal range for LCM was determined as a therapeutic target.

## 1. Introduction

Lacosamide (LCM) is a functional amino acid that is synthesized as a candidate antiseizure medication (ASM) based on the program of the National Institute of Neurological Disorders and Stroke (NINDS) in the United States, and it has been shown to be effective in multiple epilepsy animal models [1,2,3]. LCM slowly promotes sodium channel inactivation. LCM decreases neuronal hyperexcitability due to shifting the resting membrane potential further toward hyperpolarization and decreasing the fraction of available sodium channels [2]. LCM was approved in the United States and Europe in 2008 as a combination therapy for adults with focal epilepsy including secondary generalized seizures. In Japan, LCM received approval for manufacturing and marketing in 2016, and additional approval for children aged 4 years and older in 2019.

In Japan, there are many oral ASMs, but the therapeutic blood levels of recently approved drugs have generally not been established, because it is not believed that their efficacy and safety are related to blood levels [4,5,6]. There is no standard maintenance dose of LCM, because the dose of LCM can be gradually increased until patients’ convulsions are decreased or disappear. Thus, we would like to understand the pharmacokinetics of LCM for efficacy assessment, when two patients of the same weight may take different doses. However, Cawello reported that the trough concentration produces half the maximum seizure frequency reduction [7]. In clinical practice, there is some need for objective indicators to evaluate the effectiveness of LCM. Therefore, highly accurate blood levels based on strict administration management and reliable efficacy reports from patients or guardians were sought, and the correlations between oral dose and blood level and between blood level and reduction in the epileptic seizure rate were investigated. From the perspective of therapeutic drug monitoring (TDM), the peak/trough levels calculated by computer software were also examined.

## 2. Materials and Methods

### 2.1. Study Design and Ethical Considerations

This was a prospective cohort study performed according to the principles of the Declaration of Helsinki. The objective of the study and the therapeutic efficacy and safety of LCM were explained to the patients and their parents, who provided informed consent prior to enrolment. This study was approved by the Bioethics Committee of St. Marianna University School of Medicine (approval number: No. 6066). All experiments were performed according to the approved protocol.

### 2.2. Patient Selection and Treatment

This study included 101 randomly selected patients with focal epilepsy (45 females, 56 males; age range, 4.1–26.3 years) treated with LCM in the pediatric department of Kawasaki Municipal Tama Hospital and St. Marianna University School of Medicine from April 2020 to September 2022. All patients and their parents were informed about the procedure and the purpose of the study, and they all agreed to participate. The patients included 26 with focal aware seizure (FAS), 48 with focal impaired awareness seizure (FIAS), and 74 with focal to bilateral tonic–clonic seizure (FBTCS). In this study, a patient could have multiple seizure types. Details categorized by seizure type and the timing of sampling are shown in Table 1.

Eligible patients were diagnosed based on “the International League Against Epilepsy (ILAE) Commission for Classification and Terminology, 2017” [8,9] by their clinical seizure type, electroencephalogram, and either cranial computed tomography or magnetic resonance imaging. Children with other systemic (cardiac, respiratory, hepatic, renal, or endocrinological) diseases were excluded.

Before starting LCM treatment, patients received the same kinds and dosages of ASMs for 4 more weeks, but the drugs were insufficiently effective. The patients included 62 cases of monotherapy, 29 cases treated with 2 ASMs, and 10 cases treated with 3 or more ASMs. The concomitant drugs included LEV (25 cases), CBZ (18 cases), PER (7 cases), LTG (4 cases), and ZNS (3 cases). All ASMs were within the prescribed dosage. The dose of LCM was started at 1–2 mg/kg/day. If the patient showed seizures, the dose was increased by 2 mg/kg/day every 2 weeks. The maintenance dose was increased to 12 mg/kg/day for patients weighing less than 30 kg and to 8 mg/kg/day for patients weighing 30–50 kg. It was considered that the dose sufficient to eliminate seizures was the maintenance dose for each patient. For patients weighing more than 50 kg, the maximum dose was set to 400 mg/day. When LCM was added to therapy, all patients were on treatment with multiple ASMs (range, 1–3). Furthermore, the discontinuation criteria for this study were as follows: no medical record of the details of seizure patterns and frequency; no routine sampling of blood levels; no measurement of body weight at sampling time; poor adherence; and discontinuation of treatment due to serious side effects. Nine patients experienced side effects, but since their symptoms were only temporary drowsiness and resolved without intervention, they were able to continue in the study.

### 2.3. Sample Collection and Evaluation

Blood levels of LCM were measured regularly, but the time of blood sampling was any time within the consultation hours (9:00 to 16:00). The guardians were asked about the time of taking LCM in the morning, and the exact time from taking the drug to sampling blood was calculated. Blood was sampled at the pediatric outpatient clinic and measured by liquid chromatography–tandem mass spectrometry (LC-MS/MS) in an external laboratory (LSI Medience Corporation, Tokyo, Japan). A minimum sample of 0.1 mL of serum was frozen at −30 °C and saved. Blood samples for LCM level measurements were obtained at any time and were measured 1, 6, and 12 months after reaching a steady state.

The total number of blood sampling opportunities was 215. The timing of sample collection was arbitrary, so peak and trough levels were estimated individually using simulation software (PEDA VB ver.1.0.0.58).

The efficacy of LCM was evaluated by the reduction in the epileptic seizure rate (RR) at the time of blood sampling. RR at each evaluation was calculated as follows.

$$\text{Reduction in epileptic seizure rate (RR)}=\frac{B-T}{T}\times 100$$

B: paroxysmal frequency for 28 days before evaluation.
T: paroxysmal frequency for 28 days after evaluation.

Overall, a reduction in seizure frequency of greater than 50% was defined as “effective”.

### 2.4. Statistical Analysis

The correlations between parameters (LCM dose, LCM blood level, and RR) were analyzed. A statistical analysis was performed using Spearman’s rank correlation coefficient to verify the correlation and Wilcoxon’s signed-rank test for comparisons between the two groups. IBM SPSS Statistics Ver. 28.0.0.0 (190) (IBM Japan, Ltd., Tokyo, Japan) was used for all analyses.

## 3. Results

### 3.1. Details of Patients’ Age Ranges, Doses, and Blood Levels by Timing of Sampling and Seizure Type, and the Time from the Most Recent Administration to Blood Sampling

Patients’ background characteristics are shown in Table 1. The LCM dose of all cases was 5.1 ± 2.2 mg/kg/day (mean ± SD, range: 1.0–10.3 mg/kg/day), and the mean blood level was 7.1 ± 3.5 µg/mL (range: 0.5–16.0 µg/mL). There were significant differences in the LCM blood levels after a steady state was reached. Compared to the level after 1 month (6.1 ± 3.2 µg/mL), it was 8.2 ± 3.5 µg/mL after 6 months (*p* = 0.006) and 8.1 ± 3.3 µg/mL (*p* = 0.027) after 12 months. Since the blood level was relatively high, it was expected that there would be a difference in effectiveness. However, there were no significant differences. The RR was 70.8 ± 35.8% after 1 month, 75.6 ± 33.5% after 6 months (*p* = 0.539), and 73.8 ± 26.4% after 12 months (*p* = 0.145). Fortunately, only a few patients experienced side effects, including temporary drowsiness, and their symptoms improved spontaneously without them withdrawing from the study.

The doses and blood levels of the three types classified by seizure type were also examined [8,9]. Compared to the level in FAS (6.7 ± 3.2 µg/mL), the level in FIAS was 7.2 ± 3.7 µg/mL (*p* = 0.331), and the level in FBTCS was 7.1 ± 3.4 µg/mL (*p* = 0.611); there were no significant differences.

Blood samples for LCM level measurements were obtained at any time. In fact, the average time from the most recent administration to blood sampling was 2 h 53 min, ranging from 1 h 40 min to 5 h 15 min.

### 3.2. Relationship Between Dose and Blood Level

**Figure 1:**
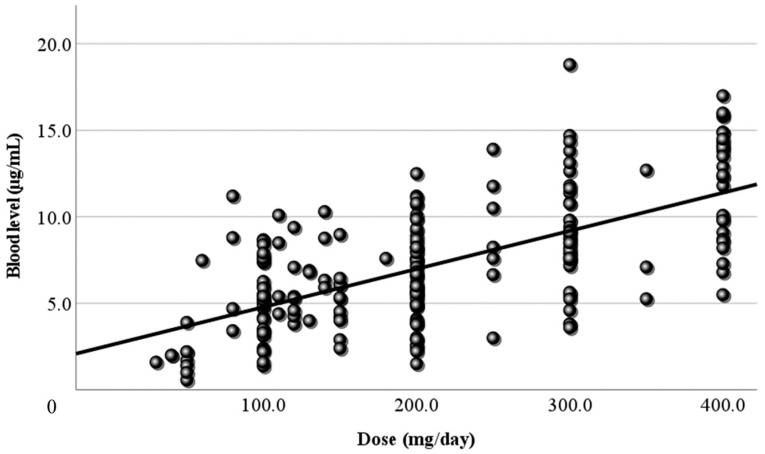
Relationship between the dose and the blood level of LCM. The daily dose (mg/day) and its blood level have a positive correlation at all sampling time points (r = 0.406). The regression line is “y = 0.02x + 2.58”.

**Figure 2:**
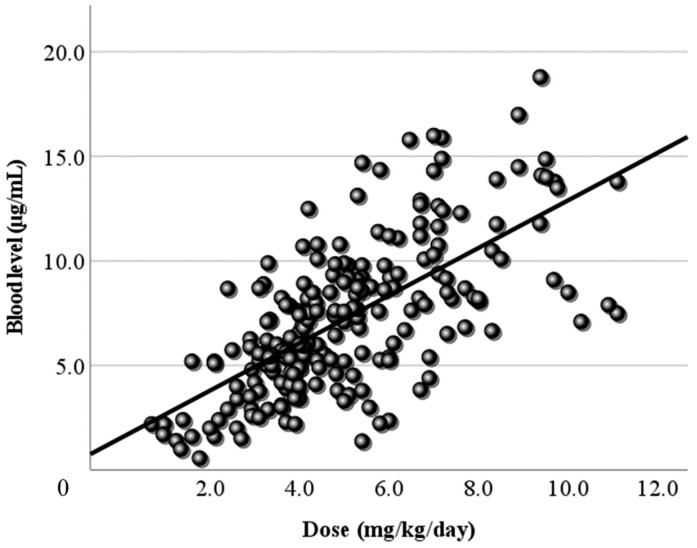
Relationship between the corrected dose and the blood level of LCM. The dose corrected by body weight (mg/kg/day) and the blood level have a positive correlation. The correlation coefficient is 0.446, slightly higher than the coefficient for the daily dose. The regression line is “y = 1.14x + 1.52”.

**Figure 3:**
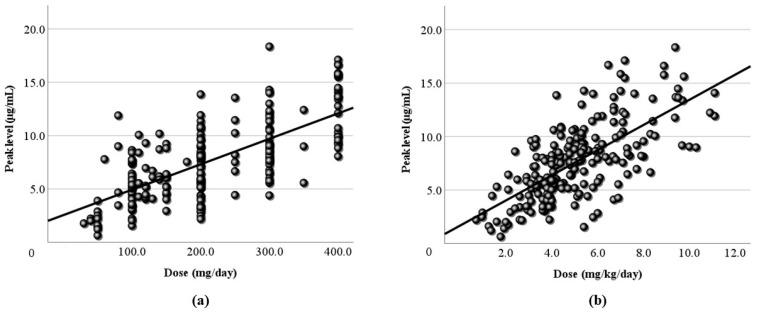
Relationship between the dose and the peak blood level of LCM. (**a**) The calculated peak blood level and the daily dose (mg/day) have a positive correlation (r = 0.479). (**b**) The peak blood level and the corrected dose (mg/kg/day) also have a positive correlation (r = 0.478). The regression lines are “y = 0.02x + 2.54” and “y = 1.18x + 1.67”, respectively.

**Figure 4:**
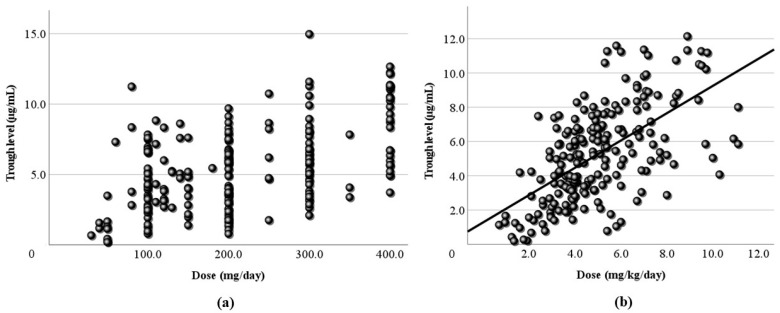
Relationship between the dose and the trough blood level of LCM. (**a**) The calculated trough blood level and the daily dose (mg/day) have no correlation. (**b**) The trough blood level and the corrected dose (mg/kg/day) have a subtle positive correlation (r = 0.372), and the regression line is “y = 0.8x + 1.27”.

Figure 1 shows the relationship between the LCM dose (mg/day) and the blood level at all sampling time points. In this study, the LCM dose (mg/day) and its blood level had a positive correlation, and the regression line was “y = 0.02x + 2.58”. However, the correlation coefficient was low (r = 0.406). When the dose was corrected by body weight, the correlation coefficient increased slightly (r = 0.446), and the regression line changed to “y = 1.14x + 1.52” (Figure 2). Moreover, the correlations between the peak/trough levels calculated by PEDA VB and the doses were also examined. The peak blood levels and doses had positive correlations (peak r = 0.479, corrected r = 0.478), and the regression lines were “y = 0.02x + 2.54” and “y = 1.18x + 1.67” (Figure 3). For the trough level, there was a positive correlation only between the trough level and the dose corrected by body weight (Figure 4) (r = 0.372, y = 0.8x + 1.27).

### 3.3. Relationship Between the Blood Level and RR

**Figure 5:**
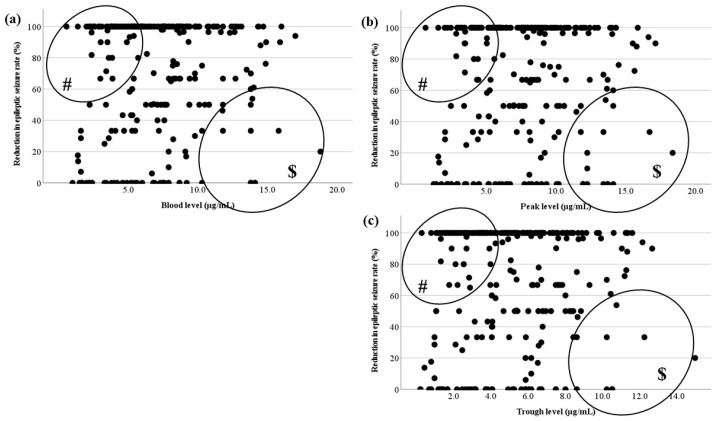
Relationship between LCM blood levels and RR. (**a**) There is no correlation between the actual LCM blood level and RR. (**b**,**c**) The calculated peak and trough levels do not correlate with RR. (#) This area includes effective cases with low blood levels; ($) another area includes the cases whose RR was not sufficient to increase with a high dose.

The relationships between the LCM blood level and RR at each timing based on TDM are shown in Figure 5. The blood levels, including actual levels, calculated peak levels, and calculated trough levels, did not correlate with RR. There were outliers. There were effective cases with low blood levels (# in Figure 5) and cases whose RR was low with a high dose ($ in Figure 5).

**Figure 6:**
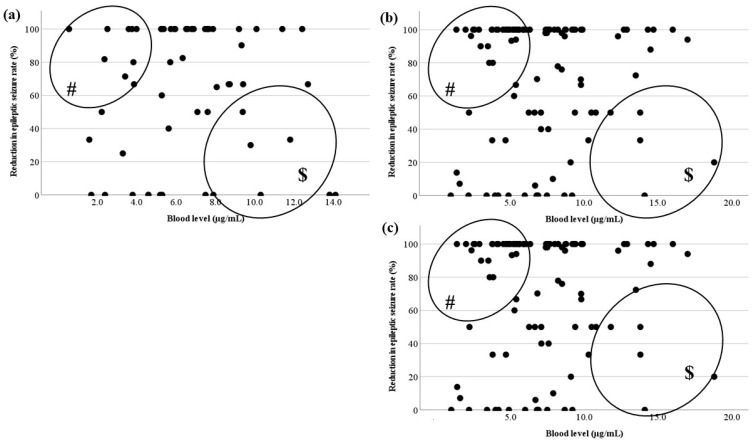
Relationship between LCM blood levels and RR for each seizure type. There is no correlation between the actual LCM blood level and RR of (**a**) FAS, (**b**) FIAS, (**c**) and FBTCS. There are effective cases whose LCM blood levels are low (#). There are low RR cases with an increased LCM dose ($).

Next, the correlation between blood level and RR was examined for three types of seizure (Figure 6). The relationships were examined in patients with FAS, FIAS, and FBTCS, but there were no correlations. There were also outliers (#, $ in Figure 6).

### 3.4. Comparisons of the LCM Blood Level Between the Effective and Ineffective Cases and Identification of the Optimal Range

**Figure 7:**
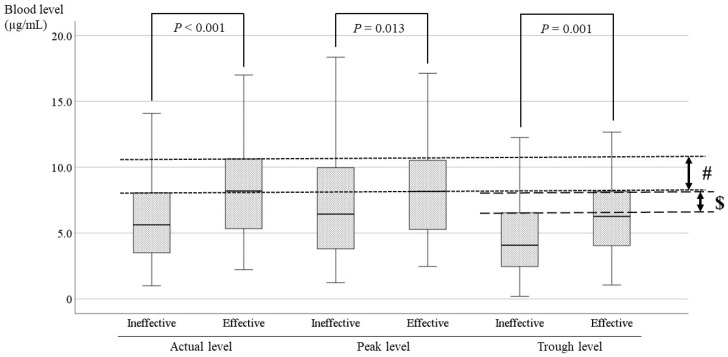
Comparisons of LCM blood levels between the effective and ineffective cases. There is a significant difference between effective and ineffective cases at all points. The optimal range encompasses the range in which the blood levels of the effective cases and the ineffective cases do not overlap. (#) For the actual level, the optimal range is 8.0–10.5 µg/mL. ($) The optimal range of the trough level is 6.5–8.0 µg/mL.

The LCM blood level was compared between effective cases and ineffective cases at points based on TDM (Figure 7). There was a significant difference between the two groups at all time points, and the blood levels of effective cases were significantly higher than those of ineffective cases (*p* < 0.04). The actual level in effective cases was 8.26 ± 3.70 µg /mL (mean ± SD), with a range of 2.2–17.0 µg/mL. Moreover, the optimal range was set to be the range in which the blood levels of the effective cases and the ineffective cases did not overlap. In the actual level that had a markedly significant difference, the optimal range was 8.0–10.5 µg/mL based on the average, standard deviation, and upper limit of the 95% confidence interval (# in Figure 7). In the same way, the optimal range of the trough level was 6.5–8.0 µg/mL ($ in Figure 7). In addition, the optimal range of the peak level was narrow, and because it overlapped with the range of the actual level, the optimal range could not be determined.

**Figure 8:**
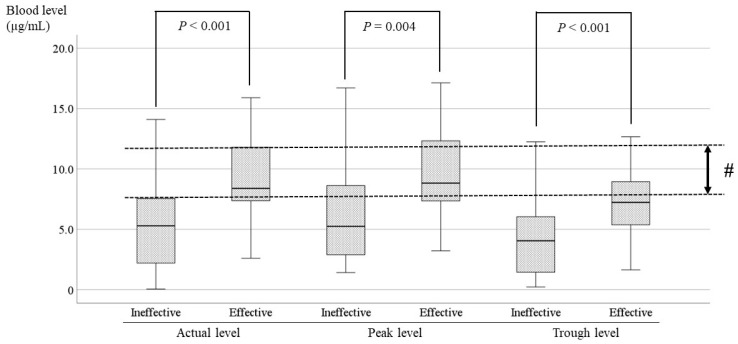
Comparisons of LCM blood levels between the effective and ineffective cases with FBTCS. The blood levels at all points of the effective cases are significantly higher than the blood levels of ineffective cases. (#) In the actual level, its optimal range is 7.5–12.0 µg/mL.

In patients with FAS and FIAS, there was no significant difference between effective and ineffective cases. Comparisons of the LCM blood levels between effective and ineffective cases with FBTCS are shown in Figure 8. In patients with FBTCS, the blood levels were significantly higher in effective cases than in ineffective cases (*p* < 0.005). The optimal ranges of the peak/trough levels could also not be set, because they almost overlapped with the range of the actual level.

## 4. Discussion

There have been many reports of the efficacy of LCM for pediatric patients [10,11,12]. Kohn suggested that measuring serum concentrations of LCM in pediatric patients during treatment might not be necessary [4]. Another reason for not measuring them may be the lack of pharmacokinetic interactions observed between LCM and other ASMs [7,13,14]. TDM is important for effective and safe treatment using oral ASMs. In our view, TDM can be helpful because of the report about CYP2C19 polymorphisms that affect the serum concentration of LCM [15].

LCM is a drug with a unique mechanism of action, slowly promoting sodium channel inactivation [2,3]. LCM has another mechanism involving the modulation of CRMP-2 activity [16,17,18]. Thus, it has been considered a promising candidate with a broad spectrum of action, including epilepsy, neuropathic pain, and other indications, and it has been adopted not only in Japan, but also in many other countries.

Though studies of LCM pharmacokinetics in pediatric patients with epilepsy have been reported [4,6,19], the therapeutic range of the blood level has not been established. There are also reports that there is no relationship between the blood level of LCM and its effectiveness [4]. Therefore, the correlations between oral dosage and blood level and between blood level and RR were investigated to try to set the therapeutic range of LCM. Unfortunately, setting the therapeutic range of LCM blood levels was difficult based on the results, because the blood level and efficacy showed no positive correlation, regardless of duration or seizure type (Figure 5 and Figure 6). However, it seemed possible to set the optimal range for the therapeutic target, because there was a significant difference between effective and ineffective cases with LCM treatment.

First, there were significant differences in LCM blood levels after a steady state was reached in the present study (Table 1). The blood level immediately after reaching a steady state was compared with levels 6 months after and 12 months after. Whereas blood levels were significantly higher 6 and 12 months after, there were no differences in effectiveness. The reasons for significant increases in doses may be due to seizure recurrence or weight gain. Whether the dose was increased without seizures decreasing, or the blood level decreased due to the patient’s growth, the RR of effectiveness seemed difficult to increase [20]. In particular, since there were many cases in which low doses of LCM were effective (# in Figure 5) [21], it was predicted that increasing the dose would be less effective in refractory cases in which seizures occurred after a steady state ($ in Figure 5). These appeared to be the reasons for the lack of correlations. No reports of the effect of LCM depending on the seizure type could be identified, but the effectiveness of LCM for generalized tonic–clonic seizures was reported [22]. Therefore, we hypothesized that its effect and the required dose may differ by seizure type, and they were compared by classifying them into three types. However, there were no significant differences in their blood levels. Thus, the hypothesis was not confirmed (Table 1). Furthermore, a correlation between blood level and RR for each seizure type was also sought, but this too could not be confirmed (Figure 6). Because it was not possible to change the oral dose or set the therapeutic range depending on the seizure type, the optimal range of LCM treatment for focal seizure was investigated.

Next, LCM doses and blood levels had a positive correlation in the present study, as in previous reports [4,6,7,23]. Both the correlation coefficient of the daily dose (Figure 1) and that of the dose corrected by body weight (Figure 2) were relatively low (r = 0.406, 0.446, respectively). Based on TDM, it was considered reasonable that the corrected dose was slightly higher than the daily dose. To set the optimal range, it was essential to demonstrate the correlation between dose and blood level. Furthermore, the peak (Figure 3) and trough levels (Figure 4) were simulated using computer software, and the correlation with dose was examined. It was found that the correlation of the peak level was slightly high, and the correlation of the trough level was low. Generally, it is thought that the peak level is more reproducible and stable than actual values, and the trough level must be more so. We thought that the sampling timing of blood caused the trough level to be scattered. It was not believed that factors such as the incorrect reporting of medication times and sudden forgetting to take medication have large effects on the trough level. Most of the collected samples were taken 2–4 h after the morning dose. These are times close to the peak level, and it appeared that even the simulated peak levels were more accurate than the trough levels.

There was no correlation between the blood level and RR in each timing based on TDM (Figure 5). As shown in Figure 6, there were no relationships in each seizure type. This could be because there were effective cases even when blood levels were low (# in Figure 5 and Figure 6), and another reason was that the RR did not increase unexpectedly, because the LCM dose was increased due to insufficient efficacy ($ in Figure 5 and Figure 6). The reason why cases with low blood levels had high efficacy was unknown, but it is a positive feature that a low dose of LCM is effective and dose-dependent side effects are prevented. Therefore, it is justified to start with a low initial dosage, since a low blood level is associated with sufficient efficacy. There is also a report that LCM treatment is effective, particularly at higher doses [19]. In the present cases, if the efficacy was inadequate at the high dose, then sufficient efficacy might not be obtained if the dose was increased to the maximum (12 mg/kg/day).

The current study showed significant differences between effective cases and ineffective cases in patients receiving LCM (*p* < 0.001, *p* = 0.013, *p* = 0.001; Figure 7). The optimal range was set to include the range in which the blood levels of the effective cases and the ineffective cases did not overlap based on the average, standard deviation, and upper limit of the 95% confidence interval. Therefore, it was suggested that the optimal range of the actual level is 8.0–10.5 µg/mL (# in Figure 7), and the optimal range of the trough level is 6.5–8.0 µg/mL ($ in Figure 7). The minimum of the ranges was established to avoid overlap with the level in ineffective cases. Because the correlation between the trough level and the dose was insufficient (r = 0.372) in the present study, the optimal recommended range is 8.0–10.5 µg/mL.

LCM has been shown to be effective in the treatment of partial seizures in patients from 4 years of age [24,25]. LCM seems to be particularly effective for tonic–clonic seizures [22]. In the present study, a significant difference was found between effective and ineffective cases of FBTCS in epilepsies with tonic–clonic seizures (Figure 8). The target range in FBTCS was slightly higher than the optimal range mentioned above. It was expected that the dosage would be adjusted proactively because the patients and their families want the seizures to be completely suppressed, and the seizures in FBTCS are easily identified by the families and have large impacts on daily life. Therefore, doctors would likely make frequent and sensitive adjustments to the LCM dose based on parental reports of detailed seizures. It was thought that the optimal ranges in FBTCS would be higher than the optimal range of all cases, and there would be less overlap between the effective group and the other group. The target range of the results in the present study was lower than existing reports [7,19,26,27,28]. There have been very few reports that advocated a range lower than the optimal range of the present study [21]. As mentioned above, there were cases in which low LCM blood levels were effective (# in Figure 5 and Figure 6), and the presence of these cases was considered one of the reasons. Furthermore, it was also considered that these cases were more numerous than the ineffective cases with higher LCM levels ($ in Figure 5 and Figure 6). This study was also conducted to determine the relationship between seizure type and LCM dose based on its blood levels [29,30], and a correlation between the LCM dose and blood level was demonstrated. However, no correlation was observed between the blood level and the RR in FAS, FIAS, and FBTCS, because there were many cases in which low doses of LCM were effective, or even high doses were insufficiently effective. Similar to the comparison of blood levels between the LCM effective group and the other groups in the whole population, the two groups were compared for each seizure type; no significant differences were observed, except for FBTCS. This might be due to inadequate medication in FAS and FIAS compared with FBTCS, due to a tendency to overlook seizures. Therefore, comparisons of the optimal ranges for each type of seizure were impossible, and the required oral dose for each type could not be stated.

Furthermore, the objective was to obtain the optimal range for many patients with various conditions. LCM is metabolized by CYP3A4, CYP2C9, and CYP2C19 in the liver, so there is concern about interactions with ASMs which are similarly metabolized. However, it has been confirmed that neither enzyme induction nor inhibition is seen with LCM. In contrast, the number of focal and generalized seizures per month, their severity, and the etiology of epilepsy are important factors, but it was not possible to quantify and evaluate them. Therefore, we did not address the effects of the interactions between ASMs, and the etiology of epilepsy was not addressed in the design of this study.

## 5. Conclusions

The present study demonstrated the efficacy of LCM and the usefulness of LCM blood level measurement. There was a positive correlation between the oral dose of LCM and its blood levels, and LCM blood levels were higher in effective cases than in ineffective cases. Therefore, the optimal range was established to be 8.0–10.5 µg/mL. Furthermore, in focal epilepsy with FBTCS, the optimal range may be expanded. The monitoring of plasma LCM levels may help physicians optimize the drug dose schedule in individual patients and lead to the easy use of LCM.

## Figures and Tables

**Table 1:** Details of patients on LCM therapy.

	Age (y)	Dose (mg/kg/day)	Blood Level (µg/mL)	^1^ *p* Value
All samples	15.3 ± 6.0	5.1 ± 2.2	7.1 ± 3.5	
Timing of sampling				
1 month	14.4 ± 6.1	4.6 ± 2.1	6.1 ± 3.2	–
6 months after	16.2 ± 6.5	5.4 ± 2.1	8.2 ± 3.5	*p* = 0.006
12 months after	15.9 ± 5.7	5.6 ± 1.9	8.1 ± 3.3	*p* = 0.027
Seizure type				
Focal aware seizure	15.7 ± 4.9	5.2 ± 2.3	6.7 ± 3.2	–
Focal impaired awareness seizure	14.9 ± 5.6	5.1 ± 2.3	7.2 ± 3.7	*p* = 0.331
Focal to bilateral tonic–clonic seizure	15.5 ± 6.6	4.9 ± 2.0	7.1 ± 3.4	*p* = 0.611

Data are the means ± SD values, ^1^ Wilcoxon signed-rank test. LCM, lacosamide.

## Data Availability

The data supporting the study findings are available upon reasonable request from the corresponding author in accordance with the data policies.
